# Optical coherence tomography complements confocal microscopy for investigation of multicellular tumour spheroids

**DOI:** 10.1038/s41598-019-47000-2

**Published:** 2019-07-22

**Authors:** Neelam Hari, Priyanka Patel, Jacqueline Ross, Kevin Hicks, Frédérique Vanholsbeeck

**Affiliations:** 1 Department of Physics, University of Auckland, Auckland, 1010 New Zealand; 2 The Dodd-Walls Centre for Photonic and Quantum Technologies, University of Auckland, Auckland, New Zealand; 3 Auckland Cancer Society Research Centre, University of Auckland, Auckland, New Zealand; 4 Biomedical Imaging Research Unit, Department of Anatomy and Medical Imaging, University of Auckland, Auckland, New Zealand

**Keywords:** Optical imaging, Imaging and sensing

## Abstract

Knowledge of optical properties, such as the refractive index (RI), of biological tissues is important in optical imaging, as they influence the distribution and propagation of light in tissue. To accurately study the response of cancerous cells to drugs, optimised imaging protocols are required. This study uses a simple custom-built spectral domain optical coherence tomography (OCT) system to conduct RI measurements of multicellular spheroids, three-dimensional (3D) *in-vitro* culture systems, of the cell line HCT116. The spheroid RIs are compared to study the effect of growth over time. To improve confocal microscopy imaging protocols, two immersion media (glycerol and ScaleView-A2) matching the spheroid RIs were trialled, with the aim to reduce the RI mismatch between the spheroid and the immersion medium and thus improve imaging depth with confocal microscopy. ScaleView-A2 (n = 1.380) aided in achieving greater depths of imaging of the multicellular spheroids under confocal microscopy. This improvement in imaging depth confirmed the utility of our RI measurements, proving the promising outlook of OCT as a complementary tool to microscopy in cancer research.

## Introduction

In cancer research, three-dimensional tumour cell cultures, also known as multicellular spheroids, serve as a biochemical and morphological *in-vitro* research model for *in-vivo* tumours^1^. In comparison to two-dimensional *in-vitro* cell models, multicellular spheroids are effective for studying cancer drug delivery with the advantage of resembling *in-vivo* tumours in terms of cellular environment with respect to oxygen and pH gradients, as well as structural heterogeneity, thus contributing to the formation of quiescent, anoxic, hypoxic, and necrotic cell subpopulations^2^. The *in-vitro* multicellular spheroid exhibits a closely-packed, spherical geometry of cells with a concentric arrangement of actively proliferating cells at the periphery, an intermediate layer of quiescent cells, and an emerging central core of dead cells deep within the spheroid^3^. The rapid multiplication of tumour cells leads to a deprivation of oxygen within the inner cells. Beyond a critical size (>500 *μ*m), most spheroids develop hypoxia leading to a necrotic core surrounded by a viable rim of cells^1^. Where these conditions prevail, they contribute to a type of cell death, necrosis.

Hypoxic regions have an important role in tumour progression and are considered a target to exploit in cancer therapy^4^. Recent studies have been conducted with spheroids to study the tumour cell response to hypoxia-activated prodrugs (HAP)^5^, which become activated at the oxygen-poor core of solid tumours. Spheroids are treated with hypoxia probes and live/dead stains to quantify the amount of hypoxia and assess the viability of cells using confocal microscopy. However, the constituents of biological tissue and lack of transparency scatter light, reducing the performance of confocal microscopy as depth into the specimen increases. In addition, refractive index (RI) mismatches limit the ability to image the entire depth of spheroids, resulting in low contrast, reduced spatial resolution with depth, and spherical aberrations^6^. Therefore in order to be able to investigate the presence of hypoxia in spheroids and evaluate drug efficacy, improvements in the ability to image intact spheroids are necessary.

Widefield fluorescence microscopy is only suitable for imaging thin samples as thicker samples result in the problem of out-of-focus blur as fluorescence from multiple planes within the sample is captured in each image by the camera thereby providing a false impression of the location of staining. Therefore, in order to determine the distribution of a fluorescent dye throughout a multicellular spheroid with widefield fluorescence microscopy, embedding and serial sectioning is required. Confocal microscopy enables imaging of thicker samples with improved resolution and contrast through its ability to acquire optical sections as only in-focus light is captured by the detector. However, confocal microscopy still has limitations, as imaging deep into samples can be problematic due to light scatter from biological structures and RI mismatch. Depth of imaging can be improved by matching the RI of the sample with the immersion medium for the objective lens, thereby substantially reducing light scattering and refraction. The ability to measure the RI of the sample allows RI matching to be achieved for confocal microscopy resulting in improved image quality and depth.

Immersion media, such as glycerol and ScaleView-A2 are examples of optical clearing agents often used in optical microscopy. Optical clearing agents can improve imaging depth by reducing the biological tissue’s scattering and absorption, and thereby increasing light transmission^7^. This is especially important in optical imaging, as it enhances the ability to visualise structures at greater depths. The decreased scattering is achieved via mechanisms such as the replacement of tissue fluid with media of higher RI, dehydration, and collagen dissociation^7^.

Another option for imaging such samples would be histological sectioning and imaging of serial sections followed by image registration and reconstruction. However, this approach is very labour-intensive and physical cutting of the samples results in information being lost. Histology was used in the current study to confirm penetration of dyes as a validation step. Another imaging technology that is suitable for such specimens is light-sheet fluorescence microscopy. However, in this case, RI matching and optical clearing would still be required.

Optical coherence tomography (OCT) is a technique which is capable of imaging the entirety of the spheroid, while also providing morphological and physiological information about it. It is label-free, using the intrinsic contrast provided by the sample, and also non-invasive, which is advantageous in the realm of cancer research. Previous studies have shown that diameter and attenuation can be measured with OCT^8,9^. Huang *et al*.^9^ conducted an impressive study with OCT detecting the necrosis within the multicellular spheroids using a label-free intrinsic optical attenuation contrast near the centre of the spheroid^9^. Furthermore, they took 3D OCT images to visualise the morphology of the spheroids and study their growth kinetics and quantify their volumes using a voxel-based approach. While this study has provided valuable information of the potential of OCT as a high-throughput imaging system, the authors have not measured the multicellular spheroid RI, which is an important parameter to consider when selecting the immersion media for microscopy.

The RI of tissue has been studied since the 1950s^10^ and is an important optical property of tissue in optical imaging techniques, such as confocal microscopy and OCT. Measurements of the RI of tissue with OCT were originally proposed by Tearney *et al*.^11^, demonstrating two methods to measure the RI of the layers of human skin^11^. The first method, the path-length matching method, extracted the RI by comparing the optical and geometrical thicknesses. The second method used OCT focus tracking, which measured the ratio between the optical path length measured by OCT and the resulting focus shift from moving the focus of the objective lens through the sample. Since then, applications based on either of these methods have been used to measure the RI of human crystalline lenses^12^, *in-vitro* human teeth^13^, and the mouse crystalline lens^14^. The path-length matching method is a simple non-invasive method which presents many advantages such as its rapid measurements, increased accuracy and easy operation, in comparison to the OCT focus-tracking method^13^.

This study investigates the RIs of multicellular spheroids and the effects of growth on HCT116 spheroids, a human colon cancer cell line. The motivation of this study is twofold. First, the spheroid RI was measured with OCT utilising the path-length matching method. This enabled the selection of an appropriate RI matched immersion liquid for confocal microscopy to reduce RI mismatch between the spheroid and immersion medium, so that greater depth of imaging through the spheroid can be achieved as less light scatter will occur. Two immersion media were chosen based on the results of the OCT RI measurements. Second, we investigated whether OCT could be used to monitor overall spheroid sizes and changes such as necrosis to provide quantitative information on spheroid growth and cell death.

## Methods

### Sample preparation

The HCT116 colon cancer cells (American type tissue culture collection (ATCC), VA, USA) were grown in a standard incubator at 37 °C with 20% oxygen and 5% carbon dioxide as monolayer cultures in T75 flasks containing *α* Minimum Essential Medium (α-MEM) (Sigma-Aldrich, USA) with 5% foetal calf serum (FCS). These cells were dissociated with trypsin and spheroids were cultured by seeding 1000 cells per well in round bottom 96 well plates (Corning, USA), consisting of surfaces that allow minimal cell attachment, encouraging the cells to form a spheroid. Every second day, the spheroids were replenished with 50% new growth medium containing α-MEM, 10% FCS and 1% penicillin with streptomycin (P/S).

The pilot study (Experiment 1) used SiHa cells (ATCC, VA, USA) prepared in the same way as above. Although SiHa spheroids were used in the pilot experiment to confirm that an OCT system could measure the RI of spheroids, HCT116 spheroids were used for the remainder of experiments as this cell line produced more tightly packed spheroids, with increased circularity and consistency in shape. The spheroids were fixed and imaged with OCT after immersion in phosphate buffered saline (PBS). Thereafter, three OCT experiments were conducted with fixed HCT116 spheroids which were immersed in ethanol (Experiment 2) or PBS (ACSRC, The University of Auckland, NZ) (Experiments 3 and 4) for 24 hours prior to OCT imaging. The fixation procedure included aspirating the media and immersing the spheroid in 10% neutral buffered formalin (NBF) for 24 hours at 4 °C, then retained in 70% histology ethanol. To compare the impact of seeding density on growth, spheroids were also prepared at a lower seeding density of 500 cells per well as well as 1000 cells per well (Experiment 4) and cultured until day 4 followed by fixation as above.

### Brightfield image acquisition and analysis

Brightfield images of live spheroids were acquired daily using the Molecular Devices ImageXpress Micro XL (Molecular Devices USA), prior to fixation. These images were used to measure growth characteristics using a macro^5,15^ (GB Spheroid Macro), developed by Gib Bogle (Auckland Bioengineering Institute, University of Auckland), for the open source software, ImageJ (NIH, USA)^5^.This macro was a modified version of one published by Ivanov *et al*.^16^. The scale was based on the system calibration (1 pixel equivalent to 0.891 *μ*m). Each image was analysed with autothresholding (Intermodes algorithm), erosion and an object detection algorithm, which enabled the spheroid to be detected, and any debris surrounding the spheroid to be excluded. The parameters measured were area and circularity (0–1, where 1 indicates a perfect circle). The area measurements calculated from this macro were then used to calculate the diameter and radius.

### OCT image acquisition and analysis

To determine the spheroid RIs, a custom-built spectral domain OCT (SD-OCT) system^17^ acquired two-dimensional images (B-scans) of the sample on a flat reflecting surface. To avoid degradation of the samples, images were taken within five minutes of removal from solution. The images of the spheroids were taken where the largest cross-sectional area could be visualised. The SD-OCT employs a 3 × 1 super-luminescent diode (Superlum, Ireland) with a central wavelength, *λ*_0_, of 840 nm, and bandwidth Δ*λ* of ≈10  nm, thus yielding a theoretical axial resolution in air of 3.1 *μ*m. The signal-to-noise ratio of the system was measured to be approximately 95 dB. The coupled lateral resolution, Δ*x*, and the depth of focus, *b*, was measured to be 21 *μ*m and 820 *μ*m, respectively. The power in the sample arm was approximately 6 mW, which did not induce any visible damage upon the sample.

**Figure 1:**
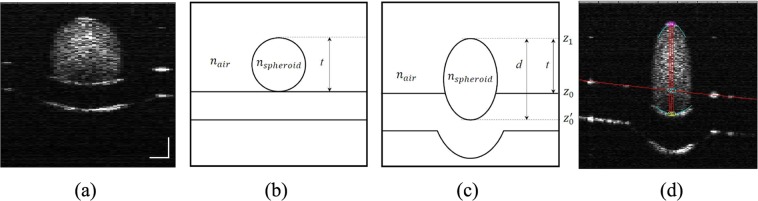
The OCT B-scan in (**a**) shows the spheroid on a Petri dish with distance calibrated in air with equal vertical and horizontal scales. Scale bar represents 200 *μ*m. Schematic representation of the spheroid thicknesses as seen physically (**b**) and by OCT (**c**). In image (**d**), the unscaled OCT B-scan in pixels of the spheroid on Petri dish show the fitted lines used to determine the geometrical thickness (*t*) and optical thickness (*d*). The circles represent the points used for the measurements of the optical thickness and the geometrical thickness: pink circles for the top surface of the spheroid, cyan circles for the approximated points of the bottom surface of the spheroid, and the yellow circles for the bottom of the spheroid in the OCT image. The red vertical lines represent the geometrical (pink to cyan) and optical thickness (pink to yellow) measurements.

OCT directly measures the group delay of light through a sample via coherence gating. A single depth scan (A-scan) in OCT represents the depth-dependent intensity of reflections or back-scattering along the beam path. The optical thickness of a sample, is measured as the distance, *d*, between reflection peaks in an A-scan, also expressed as the product of the group RI, *n*_*g*_, and the geometrical thickness, *t*, of the medium. Thus, the length of a sample depicted in an OCT image (for example, the vertical red lines in Fig. 1) is not representative of the geometrical thickness, *t*. By making an approximation of the sample’s geometrical thickness directly from the OCT image, the group RI of the samples can be determined. The group RI, *n*_*g*_, is expressed in terms of the wavelength dependence of the phase RI, *n*_*p*_, as:

1 $${n}_{g}={n}_{p}-{\lambda }_{0}{(\frac{d{n}_{p}}{d\lambda })}_{{\lambda }_{0}},$$

where *λ*_0_ is the central wavelength of the OCT light source. The $\frac{d{n}_{p}}{d\lambda }$ term is typically disregarded in practice since it is small and negligible^11,13^. Thus, we assume the group RI of the sample to be similar to the phase index.

The multicellular spheroid RI is calculated by taking measurements of the optical thickness and geometrical thickness for each individual spheroid from a single OCT B-scan (Fig. 1). The geometrical thickness is approximated as the distance from the top of the spheroid, *z*_1_, to the approximated position, achieved by polynomial fitting, of the upper reflection of the Petri dish, *z*_0_, as this path of light travels through air with a RI of 1, which is representative of the true size of the spheroid. The optical thickness is taken as the reflections from the top of the spheroid, *z*_1_, to the bottom of the spheroid, ${z}_{0}^{^{\prime} }$, as this is the path of light travelling through the sample and is influenced by the RI of the sample. The ratio of the optical thickness, *d*, to the geometrical thickness, *t*, yields the RI (Eq. 2):

2 $${n}_{s}=\frac{{z}_{1}-{z}_{0}^{^{\prime} }}{{z}_{1}-{z}_{0}}$$

OCT B-scans were taken of spheroids fixed (as described above) at days 4, 5, 6, 7 of their growth cycles for the pilot study (SiHa, Experiment 1) and three different experiments (HCT116, Experiment 2 to 4). At least three OCT B-scans were acquired for each spheroid. The images from each spheroid were analysed twice with each analysis yielding three RI measurements per image. As a result, each RI value for one spheroid is an average of at least 15 measurements. The measured RIs of the fixed spheroids from Experiment 1 (the pilot study) were used to select the immersion liquid, while the RIs from Experiment 2 to 4 were compared to study the effect of growth time.

The OCT data was corrected for background noise and the analysis is based on the assumption that the multicellular spheroid is elliptical since the circularity has been measured with the brightfield imaging to be above 90%. Also the lateral resolution of the OCT system is smaller than the cell size and does not allow us to detect the surface roughness. A MATLAB (Mathworks, USA) code, was created to semi-automate the calculation of the spheroid RI. Figure 1(d) displays the second-order polynomials which were fitted to the first reflection of the Petri dish surface (red line), and the top and bottom surfaces of the spheroid (blue line). The fittings enabled the measurements of the optical thickness and the geometrical thicknesses. The above procedure was conducted for each experiment replicate with multiple images taken of the same spheroid, thus each spheroid mean is an average of at least 15 measurements. One way ANOVA was performed on the RI dataset to determine whether there were any statistically significant differences in RI (by day or by measured spheroid size) in the data.

### Confocal microscopy image acquisition and analysis

#### Fluorescence staining of fixed spheroids

Fixed spheroids were permeabilised in PBStt (600 mL MilliQ water, 6 g Tween 20 (Global Science), 200 ml 10x PBS, 0.08 g Thimerosal (Sigma-Aldrich, USA), 0.25 g Sodium Azide (Sigma-Aldrich, USA)) for 60 minutes at 4 °C. Following the removal of PBStt, spheroids were washed with PBS and stained with either 8 *μ*M Hoechst 33342 (1 mg/mL) or 8 *μ*M propidium iodide (PI; 1 mg/mL), both prepared in PBS for a period of 24 hours at 4 °C. Stained spheroids were kept in PBS at 4 °C until required for imaging, and light exposure was minimised.

#### Image acquisition

Two different confocal microscopes were used for the study. For the initial experiments, spheroids were transferred to Ibidi 8 well *μ*-chamber slides (Ibidi, Germany) for confocal imaging so that they could be imaged using a Zeiss LSM 710 inverted confocal microscope (Carl Zeiss AG, Germany). These chamber slides have a bottom surface that is equivalent to a coverslip in both thickness and optical clarity and allowed each spheroid to be placed in a separate well and kept hydrated in PBS (n ≈ 1.33) throughout the imaging process. The objective lens used for these experiments was a 10x/0.45 NA Plan Apochromat dry objective lens with a working distance of 2 mm. For Hoechst 33342 imaging, a 405 nm diode laser was used for excitation with an emission range of 415 nm to 500 nm. For PI imaging, a 561 nm diode-pumped solid state laser was used for excitation with an emission range of 566 nm to 628 nm. A z series encompassing all of the visible staining, with a step size of 7 *μ*m and resolution of 0.415 *μ*m/pixel was collected through each spheroid.

**Figure 2:**
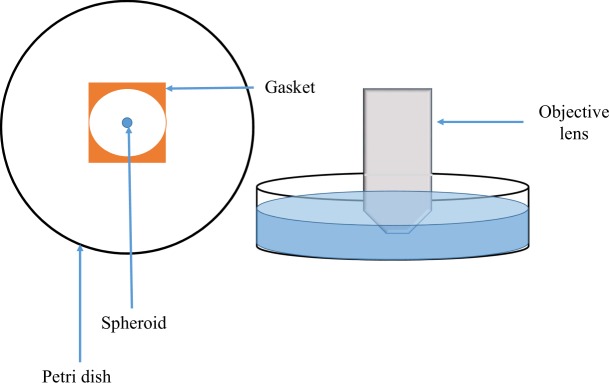
For immersion confocal imaging on the Olympus FV1000 upright confocal microscope, fixed spheroids were placed in a 90 mm Petri dish, with a droplet of mountant and sealed off by a 1 mm gasket in order to keep the spheroid stationary. The Petri dish was filled with enough immersion medium to accommodate the 10x/0.6NA XMLPV10X immersion objective lens.

For the subsequent experiments, it was determined that an upright confocal microscope would be more suitable than the inverted microscope due to the ability to use a long working distance immersion objective lens. The RI measurements determined during the pilot OCT study were used to select the optimal immersion media. Two types of immersion media, which are also optical clearing agents, were identified as being appropriate for testing: glycerol (n = 1.473; Merck, New Zealand) and ScaleView-A2 (n = 1.380; Olympus Corporation, Japan). An Olympus FV1000 upright confocal microscope (Olympus Corporation, Japan) with a specialised immersion lens (XLPLN 10x SVMP/0.6 NA) was used to image the spheroids in three different media; glycerol, ScaleView-A2 and PBS (n = 1.333). This objective lens has an adjustable correction ring enabling it to be used with a range of media with different RIs and has a working distance of 8 mm. Due to the large diameter and working distance of the objective lens, each spheroid was placed individually into a 90 mm Petri dish and sealed with a CoverWell imaging chamber gasket with built-in coverslip (Life Technologies Corporation, USA) (Fig. 2) so that the spheroid would not be affected by movement of the objective lens. Sufficient immersion medium was added to accommodate the objective lens. A z-series, with a step size of 7 *μ*m at a resolution of 0.795 *μ*m/pixel, encompassing all of the visible staining, was acquired for each spheroid. For Hoechst 33342 imaging, a 405 nm diode laser was used for excitation with an emission range of 411 nm to 461 nm. For PI imaging, a 543 nm HeNe laser was used for excitation with an emission range of 566 nm to 718 nm.

#### Image analysis

Image analysis using ImageJ was carried out on the data sets acquired on the Olympus FV1000 upright confocal microscope to compare the performance of the various immersion media. For each z-stack from representative day 5 spheroids immersed in glycerol, ScaleView-A2 or PBS, a region of interest that represented the area of the whole spheroid was created. A specialised plugin for ImageJ developed by Maske^18^ was used to derive the raw integrated density, which describes the strength of the signal and is determined by the sum of the values of the pixel within the specific area. The maximum depth of imaging (or penetration depth) was determined as the point where no fluorescence signal above background was detected, i.e., where there were no pixel values above the measured mean background value.

### Detection of necrosis in live spheroids

Necrosis in live spheroids was defined based on a protocol used by Zhang *et al*.^19^. Spheroids were seeded at 1000 cells per well. Individual spheroids were removed on days 4, 5, 6, 7 and 9 with 100 *μ*L of media and placed into Eppendorf tubes with 6 *μ*L of PI (1 mg/mL)(8 *μ*M in PBS). Tubes were incubated at 4 °C for 90 minutes, after which spheroids were replaced back into wells with fresh culture media, *α*-MEM +10% FCS +1% P/S. Live spheroids were imaged on the ImageXpress, using the Triple 2 (TRITC) filter set with excitation 549/15 nm and emission 580–648 nm wavelengths.

## Results

### RI quantification of spheroids

**Figure 3:**
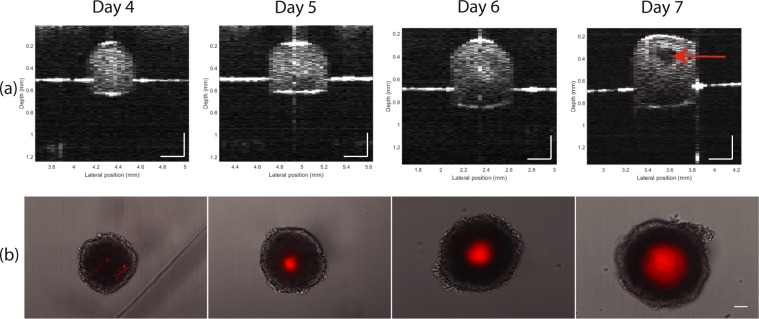
(**a**) Evolution of HCT116 spheroid structure as imaged by OCT. Cross-sectional OCT images showing the growth of the spheroids with day and the presence of necrosis (red arrow) at day 7. Scale bars on OCT images represent 200 *μ*m. (**b**) Brightfield images of HCT116 spheroids merged with widefield fluorescence imaging of the PI identifying dead cells in the necrotic core. Scale bar on images represent 100 *μ*m.

Representative spheroids visualised with OCT and their corresponding merged brightfield and fluorescence microscope images are shown in Fig. 3a and b, respectively. The OCT images display the full depth of the fixed spheroid showing elliptical morphology. OCT images of spheroids greater than day 5 display clear dark regions near the centre of the spheroids (Day 7 in Fig. 3), corresponding to regions of necrosis, due to the lower backscattering from the lack of structure, thus decreasing the OCT signal. However, near the bottom of the spheroid, the lack of OCT signal intensity present is due to higher OCT signal attenuation and is not representative of necrosis.

**Figure 4:**
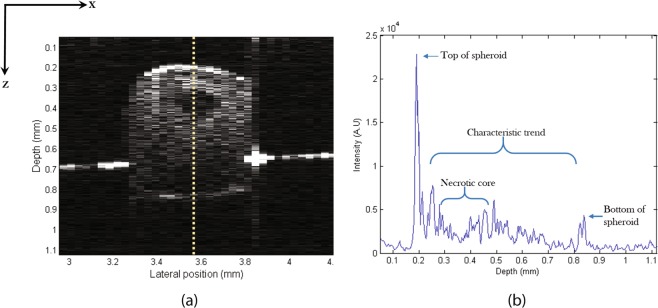
(**a**) B-scan of HCT116 spheroid at day 7. (**b**) Depth scan profile taken at x = 3.56 mm. A dip in the profile between 0.3 mm and 0.5 mm coincides with the dark necrotic region in the B-scan on the left.

Figure 4, which displays an A-scan passing near the centre of the spheroid, exemplifies the fluctuations in signal with depth as well as the attenuation of the signal with depth, which is a typical characteristic with OCT A-scans, before detection of a major reflection of the bottom of the spheroid at approximately 0.85 mm. The dip in signal between 0.3 mm and 0.5 mm corresponds to the signal from the necrotic core.

**Figure 5:**
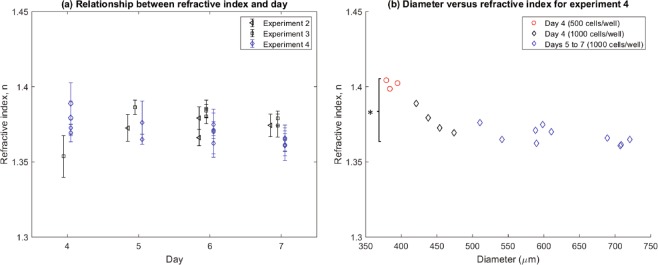
(**a**) Measured RIs (n ± *σ*) for each replicate at each day excluding Experiment 1 (Pilot study which used the SiHa cell line). Each point represents the mean and standard deviation of at least 15 measurements performed for each HCT116 spheroid. All spheroids in this graph were cultured with a seeding density of 1000 cells per well. (**b**) Dependence of RI on spheroid diameter: To compare the effect of seeding density and spheroid diameter on RI, Experiment 4 also included spheroids seeded at 500 cells/well. Spheroid diameter was measured with live spheroid imaging.

The average RI was quantified through the centre of each spheroid (Fig. 5(a)). The results from the pilot study (Experiment 1) with SiHa cells gave measurements of the RI ranging from n = 1.36 to 1.47. These results allowed us to select and test appropriate immersion media for the study. The measured RIs for HCT116 spheroids seeded at 1000 cells per well (n = 1.35 to 1.39) show no significant difference between days (p-value > 0.05; One-way ANOVA), despite the onset of necrosis visible at day 7 by OCT. Figure 5(b) shows that the RI values of day 4 spheroids seeded at 500 cells per well (n ≈ 1.39) are significantly higher than spheroids from the same experiment seeded at 1000 cells per well (n ≈ 1.37) (p-value < 0.05; One-way ANOVA). Figure 5(b) shows the decreasing RI with increasing spheroid size as measured by live spheroid imaging for Experiment 4.

### Immersion media matching

**Figure 6:**
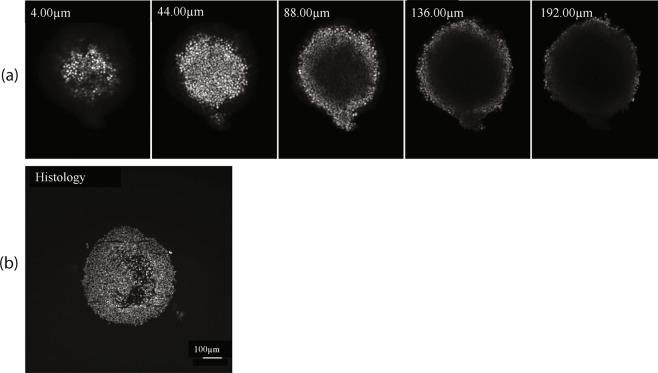
(**a**) SiHa spheroids were cultured with a seeding density of 1000 cells per well and were fixed in 10% NBF at 4 °C for 24 hours, followed by transfer into 70% histology ethanol. Fixed spheroids were exposed to Hoechst 33342 (8 *μ*M) overnight. An imaging depth of 192 *μ*m (step size = 4 *μ*m) was achieved for a day 6 SiHa spheroid stained with Hoechst 33342 for 24 hours using the Zeiss LSM 710 inverted confocal microscope, 10x/0.45NA Plan Apochromat dry objective lens, and 405 nm diode. The images are from a z-series of a representative day 6 SiHa spheroid showing the early loss of signal, particularly in the central region. The z position of each image is displayed in the corner. (**b**) Image of a central transverse histological section from a similar spheroid stained with Hoechst 33342 for 24 hours before sectioning, showing confirmation of a complete dye penetration and the presence of a thick viable rim which is not visible with confocal microscopy. The apparent gap is not due to lack of staining as adjacent cells are well stained. It is most likely due to necrosis as this was a day 7 spheroid. Additionally, this necrotic area is also off-centre thus confirming it is not an issue of restricted dye penetration through deeper central regions.

Initially when PBS (n ≈ 1.33) was used as an immersion medium in conjunction with confocal imaging on a Zeiss 710 LSM and a dry lens, the confocal microscopy setup produced 3D images of the spheroids with very limited imaging depth. Figure 6 shows that at a depth of 192 *μ*m, there was very little signal apparent. As demonstrated by the z-series shown in Fig. 6, a central dark area devoid of staining was observed. However, histological analysis of serial sections confirmed that 24 hour incubation with Hoechst 33342 stained the entire spheroid. A viable rim was identified which was much thicker than demonstrated by confocal microscopy, indicating an issue with light penetration, rather than lack of staining in the centre.

The RI measurements aided in improving the depth of imaging of the spheroids with confocal microscopy. In particular, the day 4 and 5 spheroids, when less or no necrosis was visualised, as demonstrated by PI staining (Fig. 3), were valuable for testing the two immersion media selected for trial. To improve the imaging conditions, the knowledge of the spheroid RI obtained from the OCT measurements allowed us to select appropriate immersion media that minimised the mismatch in RI. Two immersion media, glycerol (n = 1.473) and ScaleView-A2 (n = 1.380), matching the spheroids RIs were trialled with confocal microscopy. ScaleView-A2 was chosen as the results from the OCT measurements indicated a RI between 1.35 and 1.39. However, the pilot OCT study with SiHa cells reported higher RIs at day 6 of 1.43 and 1.46, which indicate that glycerol, despite its higher RI than the HCT116 spheroid RI, might be an appropriate immersion medium. Moreover, glycerol is a commonly used optical clearing agent and therefore provides a good comparison with previous studies. The protocol was further improved with the use of a specialised immersion lens. Both glycerol and ScaleView-A2, when employed with the specialised immersion lens, enabled better imaging depth than the initial immersion liquid PBS (Table 1).

**Table 1:** Depth of z-stacks, indicative of imaging depth, achieved in confocal microscopy with different immersion liquids, PBS, glycerol, and ScaleView-A2.

Depth achieved (*μ*m)				
	Propidium Iodide		Hoechst 33342	
	Mean	SD (N)	Mean	SD (N)
PBS (n ≈1.33)	220	25 (8)	188	20 (6)
Glycerol (n = 1.473)	440	− (2)	520	80 (7)
ScaleView A2 (n = 1.380)	444	34 (5)	424	0 (1)

Spheroids were seeded at 1000 cells per well and grown for 4 or 5 days. The depth varied with dyes: Propidium Iodide and Hoechst 33342. Microscopy was performed on the Olympus FV1000 confocal microscope.

**Figure 7:**
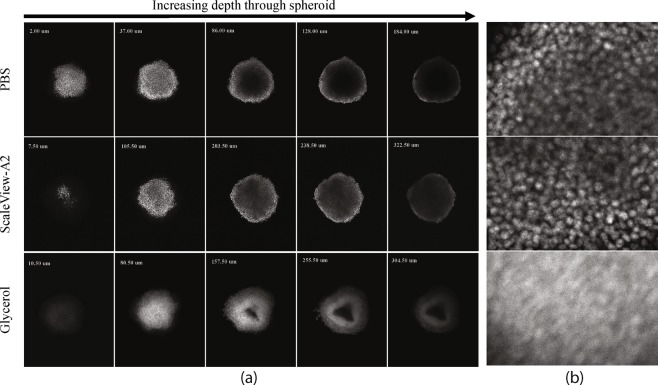
(**a**) Single confocal optical sections from a z-series of Hoechst 33342 stained HCT116 spheroids immersed in PBS, ScaleView-A2 or glycerol. The z position of each image is displayed in the corner. HCT116 spheroids, seeded at a density of 1000 cells per well, were fixed on day 5 in 10% NBF. They were stained with Hoechst 33342 (8 *μ*M) for a period of 24 hours at 4 °C. Spheroids were then kept in either ScaleView-A2 or glycerol for a period of 24 hours prior to being imaged on the Olympus FV1000 confocal microscope, using the immersion objective (XLPN10XSVMP), 10x/0.6NA. (**b**) Close-up of PBS at 128 *μ*m, ScaleView-A2 at 238.50 *μ*m and glycerol at 255.50 *μ*m.

When spheroids were imaged while immersed in PBS, the depth of imaging was very restricted as demonstrated by the early onset of signal attenuation (Fig. 7, top) with a total imaging depth of only 180–220 *μ*m. A central dark area devoid of staining was observed although histological analysis had previously confirmed that 24 hour incubation with Hoechst 33342 stained the entire spheroid and that there were cells present in the centre.

**Figure 8:**
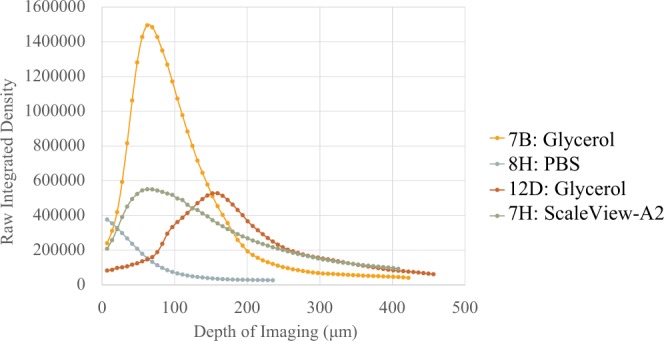
Graph showing mean signal through a z-series for individual spheroids immersed in different media. HCT116 spheroids, seeded at a density of 1000 cells per well were fixed on day 5 in 10% NBF. They were stained with Hoechst 33342 (8 *μ*M) for a period of 24 hours at 4 °C. Spheroids (excluding those in PBS) were then kept in either ScaleView-A2 or glycerol for a period of 24 hours prior to being imaged on the Olympus FV1000 confocal microscope, using the immersion objective (XLPN10XSVMP), 10x/0.6NA. Each point represents a measurement in the z-series.

Glycerol enabled a greater penetration depth of at least 413 *μ*m (Table 1). However, in comparison to ScaleView-A2 and PBS, the structural integrity was compromised and the individual nuclei were less distinct. A central dark area was evident when glycerol immersion was employed and for both PBS and glycerol immersion, the profile of the Hoechst 33342 stain was not uniform, making it initially difficult to discern whether the central dark area was due to necrosis or limited penetration of the stain into the spheroid (Fig. 7, middle). In terms of signal intensity or raw integrated density, glycerol showed inconsistency with a peak and sharp decrease, in addition to two different curves (Fig. 8).

ScaleView-A2 provided a better RI match to the spheroid RI measurements as determined by OCT. Although the penetration depth achieved by ScaleView-A2 was not greater than that achieved with glycerol, the individual nuclei were better resolved (Fig. 7). Moreover, the visualisation of the staining was more uniform with a more gradual decrease in signal intensity throughout the spheroid than with glycerol, and the structural integrity of the spheroid was not compromised.

### Diameter measurements

**Figure 9:**
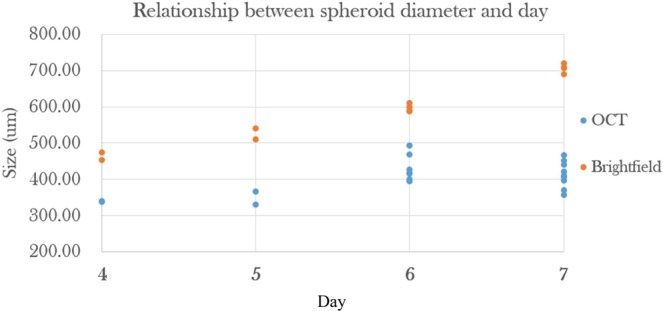
Growth of the HCT116 spheroids used in this study as a function of time, as measured from brightfield images (spheroid diameter) of live spheroids using the ImageXpress. For comparison, measurements on the same spheroids made by OCT (geometrical thickness, *t*) are shown.

Measurements of spheroid diameter by brightfield microscopy allowed comparison with measurements by OCT (geometrical thickness, *t*). The trends are similar for the diameter values obtained by OCT but lower by about 30% (Fig. 9), presumably due to shrinkage during fixation. The circularity value was always above 0.9 which would only account for a 10% diameter difference not 30%.

## Discussion

The images and measurements taken with OCT open up many possibilities for the study of multicellular spheroids in cancer research. It is important to note that OCT allows us to visualise the full depth of the spheroids in comparison to confocal microscopy. With OCT, we measure the RI of a multicellular structure averaged along the path of light through the centre of the spheroid. The measurement is a result of a combination of different cells, possibly at different stages of growth. When extending this to a multicellular spheroid, we can assume a model where, as growth occurs, there is a development of different cell populations within, normal (viable) cells near the periphery, necrotic cells (dead) at the centre, and apoptotic cells (dead) distributed throughout^20^. As a result, RI values of the spheroids reflect typical values of cellular component RIs, which range from n = 1.355 for the nucleus to n = 1.600 for lysosomes as summarised by Liu *et al*.^10^.

There is a clear size dependence of the RI for HCT116 spheroids. OCT measured a higher RI for smaller spheroids (seeded at 500 cells per well) in comparison to the large spheroids (1000 cells per well) in the same experiment. The overall higher RI displayed by the small spheroids reflects a combination of a change in the individual cell’s composition at the subcellular scale and the proportion of different cells at the multicellular scale. As stated by Liu *et al*.^10^, the RI of a cell can be used to monitor the stage of the individual cell growth cycle^10^, as the amount of intracellular components changes at each phase of growth. For example, there is an increase in RI of the cell when the DNA content within the nucleus doubles, which coincides with the transition of the cell from earlier cell growth stages of G1/S to later stages of G2/M^21^. However, many human cancer cells display atypical cell cycles and increase in cell proliferation^10^ which may contribute to the higher RI. Previous studies have measured the RI of normal and cancer cells and have confirmed RIs of 1.353 for normal cells and a range from 1.370 to 1.400 for cancer cells^22,23^. In comparison to normal cells, cancerous cells contain a larger concentration of protein (n = 1.50–1.58) in the nucleus and thus display a greater RI to normal cells^22^. We hypothesise that a contribution to the higher RI may potentially be when more cells of the spheroid are in the G2/M phase of the cell cycle, which consists of a higher nuclear content.

Cell death generally occurs via two different pathways, apoptosis and necrosis^24^. Apoptosis describes a natural programmed pathway of cell death, while necrosis is often called “accidental” cell death, as it occurs when cells are exposed to extreme variance in conditions, whether thermal, chemical or anoxic. The different processes influence the intracellular water in different ways, where apoptosis is characterised by water loss, while necrotic cells absorb water^25^. Furthermore, apoptosis occurs at any stage of growth and is not confined to only the centre of the spheroid. The current study confirmed that changes in RI were evident throughout the spheroid. With further analysis such as image segmentation and curve fitting, the RI of the individual components of the spheroids can be measured.

The difference in the range of spheroid RIs between the pilot study SiHa and the HCT116 cells may be attributed to the fact that the characteristics of multicellular spheroids vary with the origin and type of cell line used^15,26^. However, one set of measurements of the SiHa spheroids was not sufficient to confirm the difference, but provided guidance for the rest of the study. Over days 4 to 7 of spheroid growth, the cells rapidly multiplied and the spheroids grew in size, as expected^15^. However, the spheroid RIs did not show a trend over that short period of time, which indicates the need for a longer longitudinal study. We must point out that the error on the RI measurements (0.01) is mainly due to the small sample size and the low lateral resolution. Future studies should include a bigger sample size and use a system with a better lateral resolution.

The discrepancy between the diameter measurements obtained by OCT and confocal microscopy is possibly due to spheroid shrinkage during the fixation process. The increased discrepancy at day 7 (Fig. 9) may be attributed to the larger proportion of necrotic debris present compared to earlier time points which might influence the shrinkage process during fixation. It could also be corrected by a 3D measurement to ensure the diameter is measured exactly at the centre of the spheroid.

Initially, the use of PBS with a dry objective lens, introduced a mismatch between two major interfaces: air/PBS (*n* = 1.00/*n* ≈ 1.33) and PBS/spheroid (*n* ≈ 1.33/*n* = 1.35–1.39) resulting in a very shallow imaging depth. Using an immersion lens in PBS did not greatly improve the imaging depth of imaging, which indicated that there was still an issue with light penetration, probably due to the difference in RI between PBS and the spheroid causing light scattering due to refraction at the interface. With the application of glycerol (n = 1.47) or ScaleView-A2 (n = 1.38) in conjunction with the immersion lens, the mismatch was reduced to one interface: media/spheroid. This likely lessened the spherical aberration with depth compared to the original data set. To reduce the mismatch at the immersion liquid/sample interface, the RI of the cells near the cell/liquid interface were theoretically important as the RI mismatches on a macroscopic scale, e.g., between spheroid and immersion medium, determine the refraction of light.

Glycerol appeared to perform better in terms of optical clearing, as a greater depth of imaging was achieved and the spheroid was more transparent, however the distorted shape, less visible nuclear details, and inhomogenous staining proved to be drawbacks. When fixing spheroids for microscopic studies, the damaged cell membrane becomes permeable to dyes, and thus the Hoechst 33242 dye is able to penetrate into the centre of the spheroid. However, the dye, which stains dead cells and the DNA in cell debris, was not seen in confocal images of the centre of the spheroid, when immersed in glycerol. In contrast, when ScaleView-A2, which matched the spheroid RI better than glycerol, was used homogeneous staining was clearly seen at equivalent depths, which was consistent with histological sections, although the depth of imaging achieved was slightly reduced.

We have demonstrated that, after day 5, the core of the spheroid is not cellular, but consists of Hoechst 33342 staining cellular debris^15^. A perfect RI match enables no contrast with transmitted light microscopy with the spheroid appearing invisible. All the mechanisms involved in the improved imaging depth achieved when using ScaleView-A2 are not yet completely understood for this particular tissue type, but is likely to be due to the combination of (1) reduction of RI mismatch and (2) effects of the optical clearing process. These two effects could not be distinguished separately, however the knowledge gained from this study will be applied to future studies of whole spheroid imaging of hypoxia by confocal microscopy.

OCT has advantages such as its fast image acquisition time, 3D imaging, full depth visualisation of the spheroids and non-destructive and non-invasive technology. This study identified improvements that should be made such as the need to remove the spheroid from solution before imaging under OCT and fixation of spheroids. Future work will include imaging live spheroids as recently described^9^ in wells without fixation. Furthermore, lessening the power of the OCT imaging beam upon the sample (6 mW in this study) will ensure no damage to the sample while imaging. This can furthermore be resolved by imaging the spheroid while submerged in solution. However, strong reflection from the immersion fluid does influence image quality, so the spheroid must be covered in sufficient fluid, so that the air/fluid interface is outside the coherence gate when imaging. In addition, the ideal imaging system includes imaging the spheroids *in-vitro* i.e. without fixation which could be easily achieved with a portable system including a handheld probe that can be used where the spheroids are cultured. This portable system would allow us to minimise any disturbances due to removal of spheroids from their standard growth conditions when using the OCT system to help determine or predict any cellular changes that would occur in the spheroid, either naturally or as a result of treatment. Future applications will incorporate both fixed and fresh spheroids.

Our results highlight that RI has the potential to be used as a marker for spheroid growth phase. The increase in RI in small spheroids implies that variations in the RI of cells can provide more information on growth conditions. In this study, OCT could not provide sufficient information on the ratio of necrosis to proliferating cells in the spheroid, so direct inferences were not possible. The necrosis visualised in the OCT structural images (Fig. 3a, Day 7) is due to a change in RI, which demonstrates the importance of light attenuation (Fig. 8) as a marker of necrosis. Huang’s study confirmed the potential of necrosis being identified through different attenuation coefficients by necrotic cells within the spheroid. OCT allows the quantitative measurement of the local optical attenuation coefficient, *μ*_*t*_, which is described as the decay in intensity against depth. It would be interesting to see the changes in *μ*_*t*_ by day. It shows the potential of OCT for necrotic core visualisation without dyes (attenuation coefficient). Furthermore, a study by van Leeuwen *et al*. (2010) demonstrated measurements of the attenuation coefficients in viable, apoptotic and necrotic cells^27^. This supplementary information of the spheroid is advantageous when distinguishing between the two cell deaths as there is potential for necrotic core visualisation without dyes using the attenuation coefficient.

At a broader level, information on the RI, gained from OCT, provides increased knowledge about the characteristics of multicellular spheroids. This information, when using multicellular spheroid models may complement imaging techniques such as OCT and confocal microscopy. Anything we can add to further improve our knowledge regarding spheroids is beneficial as they are an *in-vitro* model with greater clinical relevance than monolayer cell cultures. This additional information may help in achieving shortened drug discovery timelines, reducing cost of investment, and bringing new medicines to patients sooner.

## Conclusion

In conclusion, with a simple custom-built SD-OCT, we successfully conducted RI measurements of large (n = 1.35–1.39) and small spheroids (n ≈ 1.39–1.41) non-invasively and non-destructively with rapid image acquisition. The variation between the spheroids at each day can be attributed to both the change in cell composition and the proportion of live/dead cells. These results suggest that alterations in RI may be a sensitive indicator of changes resulting from growth or treatment. OCT also allows visualisation of necrosis when established. Improving the OCT resolution would allow earlier detection of necrosis and such optical coherence microscopy is an imaging facility worth investigating. Furthermore, the findings were directly implemented in confocal microscopy to reduce the RI mismatch between the spheroid and the immersion medium. ScaleView-A2 aided in achieving greater imaging depths of the multicellular spheroids under confocal microscopy. This improvement in imaging depth confirmed the utility of our RI measurements, proving the promising outlook of OCT in cancer research.

## Data Availability

The datasets generated during and analysed during the current study are available from the corresponding author on reasonable request.
